# Regulating Leaf Photosynthesis and Soil Microorganisms through Controlled-Release Nitrogen Fertilizer Can Effectively Alleviate the Stress of Elevated Ambient Ozone on Winter Wheat

**DOI:** 10.3390/ijms25179381

**Published:** 2024-08-29

**Authors:** Nanyan Zhu, Yinsen Qian, Lingqi Song, Qiaoqiao Yu, Haijun Sheng, Ying Li, Xinkai Zhu

**Affiliations:** 1 College of Animal Science and Technology, Yangzhou University, Yangzhou 225000, China; dx120200148@stu.yzu.edu.cn; 2 College of JunCao Science and Ecology (College of Carbon Neutrality), Fujian Agriculture and Forestry University, Fuzhou 350002, China; 3 Jiangsu Key Laboratory of Crop Genomics and Physiology/Jiangsu Key Laboratory of Crop Cultivation and Physiology, College of Agricultural, Yangzhou University, Yangzhou 225000, China; dx120220114@stu.yzu.edu.cn (Y.Q.); 15046086520@163.com (Q.Y.); 4 College of Environmental Science and Engineering, Yangzhou University, Yangzhou 225009, China; slq231010@163.com (L.S.); hjsheng@yzu.edu.cn (H.S.); 5 Department of Biology, Hong Kong Baptist University, Hong Kong 999077, China; 6 Jiangsu Co-Innovation Center for Modern Production Technology of Grain Crops, Yangzhou University, Yangzhou 225009, China; 7 Joint International Research Laboratory of Agriculture and Agri-Product Safety, The Ministry of Education of China, Yangzhou University, Yangzhou 225009, China

**Keywords:** ozone, controlled-release nitrogen fertilizer, flowering period (Z60), flag leaf, soil microorganisms, nitrogen-cycling

## Abstract

The mitigation mechanisms of a kind of controlled-release nitrogen fertilizer (sulfur-coated controlled-release nitrogen fertilizer, SCNF) in response to O_3_ stress on a winter wheat (*Triticum aestivum* L.) variety (Nongmai-88) were studied in crop physiology and soil biology through the ozone-free-air controlled enrichment (O_3_-FACE) simulation platform and soil microbial metagenomics. The results showed that SCNF could not delay the O_3_-induced leaf senescence of winter wheat but could enhance the leaf size and photosynthetic function of flag leaves, increase the accumulation of nutrient elements, and lay the foundation for yield by regulating the release rate of nitrogen (N). By regulating the soil environment, SCNF could maintain the diversity and stability of soil bacterial and archaeal communities, but there was no obvious interaction with the soil fungal community. By alleviating the inhibition effects of O_3_ on N-cycling-related genes (*ko00910*) of soil microorganisms, SCNF improved the activities of related enzymes and might have great potential in improving soil N retention. The results demonstrated the ability of SCNF to improve leaf photosynthetic function and increase crop yield under O_3_-polluted conditions in the farmland ecosystem, which may become an effective nitrogen fertilizer management measure to cope with the elevated ambient O_3_ and achieve sustainable production.

## 1. Introduction

The atmospheric photochemical pollutant ozone (O_3_) possesses potent oxidation and toxic effects on plant growth and production [1]. In recent years, the widespread utilization of fossil fuels has resulted in a global rise in ambient O_3_ concentration [2]. Currently, we are confronted with a critical situation characterized by heightened levels of ambient O_3_ [3]. It has been reported that the ambient concentration of O_3_ in China has reached 41 ppb and is rising at a rate of 3 ppb per year. Particularly, in economically developed regions like Jiangsu Province, it has escalated to levels between 60 and 70 ppb, posing a significant threat to agricultural production and food security [4].

Up to now, the stress effects of elevated ambient O_3_ on plant apparent traits [5], soil nutrient cycling processes [6], and soil microbial communities [7] have been extensively investigated. Relevant studies have demonstrated that O_3_ inhibits crop growth and biomass accumulation, affecting crop quality and yield [8,9], and ultimately reducing the carbon sequestration capacity of farmland ecosystems [10]. O_3_ directly acts on plant leaves and inhibits photosynthesis, which will reduce the chlorophyll content and stomatal conductance of leaves [11]; and indirectly affects the underground process of plants by reducing the carbon content in root exudates and changing the types of root exudates [12]. In addition, O_3_ can also change the soil’s physical and chemical properties, affect the circulation of nutrients and the activity of related enzymes, and disrupt the stability of microbial community structure by inhibiting the nutrient and energy supply from the environment to microorganisms [13].

Soil microorganisms provide power and guarantee nutrient cycling and energy flow between plants and soil by participating in the decomposition and transformation of soil nutrients, which is an important part of the farmland ecosystem [14]. More importantly, due to the sensitivity of soil microorganisms to environmental changes, changes in microbial community structure and metabolic processes occur before plants exhibit observable O_3_ stress symptoms [15], which is of great value for exploring O_3_ mitigation options for crop production stress. However, previous studies on soil microbial communities under O_3_ stress have mostly focused on soil bacterial and fungal communities [7,16], and the research on soil archaea is still limited. Compared with the soil bacterial community, although the abundance of soil archaeal community soil is quite low, it is involved in important processes and plays a vital role in the soil nutrient cycling processes [17,18,19]. Therefore, it is a potential soil nutrient pool [20] and also an important topic of soil microbial research. The emergence of metagenomics provides a new perspective for analyzing the responses of soil microbial communities to various environmental stresses. The metagenomic sequencing process can omit the separation and purification process of microorganisms and simultaneously perform the sequencing and assembly of soil bacteria, fungi, and archaea [21], intuitively showing the differences in soil microbial community structure under different field management modes. Moreover, the sequences obtained by metagenomic sequencing can be compared with the Kyoto Encyclopedia of Genes and Genomes (KEGG) and enzyme databases to further explore the changes in metabolic functions of microorganisms from the perspective of functional genes and enzymes [22].

The current research on how to alleviate the impact of ambient O_3_ on agricultural production primarily focuses on optimizing crop varieties to improve resistance and tolerance toward ambient O_3_ [23], regulating water and fertilizer management to optimize the growth environment [24], and using exogenous chemical protectants to enhance the antioxidant capacity [25,26]. The research on the impacts of O_3_ stress on crop production and soil microbial function, as well as the mechanisms underlying various mitigation measures, remains insufficiently comprehensive. O_3_ is mainly absorbed into the crops through the stomata on the leaves, so the leaves are the initial sensor of crops under O_3_ stress and the entry point to alleviate its effects [11]. The feasibility of controlled-release nitrogen fertilizer alleviating O_3_ stress is that controlling the release rate of nitrogen fertilizer to coordinate the supply of soil nitrogen may alleviate the decrease in chlorophyll and leaf area and reduce the inhibition of O_3_ on photosynthesis of crop leaves [27]. Sulfur-coated controlled-release nitrogen fertilizer (SCNF) is a long-acting nitrogen fertilizer that delays the release rate of N elements. It is considered to be an effective product to reduce N loss and field management pressure by controlling the balance between the supply rate of soil inorganic-N and the absorption demand of crops [28,29]. Previous studies have shown that the twice-split application of SCNF can improve crop yield and N use efficiency while reducing the fertilization times compared to the multiple application of urea in conventional planting management [30]. Therefore, we hypothesized that substituting SCNF for urea, which is commonly used today, may alleviate the inhibitory effect of elevated ambient O_3_ on farmland ecosystems.

Wheat (*Triticum aestivum* L.) is a typical O_3_-sensitive crop in the world’s important food crops [31]. According to statistics, from 2014 to 2019, the loss of wheat yield caused by ambient O_3_ stress in the Yangtze River Delta region accounted for about 20% of the actual production [32]. The reduction in wheat yield caused by elevated ambient O_3_ is one of the important problems affecting world food security that needs to be solved urgently [33]. Some reports have shown that photosynthesis is the physiological basis of high yield of crops, and the contribution of photosynthetic products of functional leaves to yield can reach 70–80% in the late growth period of wheat [34]. The flag leaf is the first leaf below a wheat ear. Although the shortest leaf age and smallest leaf area, the flag leaf contains more chlorophyll than others, which directly affects the accumulation of carbohydrates in wheat grains and plays a decisive role in yield [35]. Therefore, it is of great significance to alleviate the effect of O_3_ on the morphology and physiology of flag leaves and maintain photosynthetic function to stabilize wheat yield.

In summary, it is of great theoretical and practical significance to carry out research on the responses and mitigation technologies to the elevated ambient O_3_ in typical farmland ecosystems in Jiangsu Province and to clarify the effects of SCNF on wheat production and soil biological characteristics under O_3_ stress. We hypothesized that, by regulating the N supply to the soil, SCNF may regulate the morphological and physiological characteristics of wheat as well as the physical and chemical properties of soil, thereby mitigating the wheat yield loss caused by the O_3_-stressed environment. In this study, ozone-free-air controlled enrichment (O_3_-FACE) facilities and meteorological monitoring devices were used to simulate the scenario of elevated ambient O_3_, and field experiments were conducted to study the effects of SCNF instead of urea on the photosynthetic function of wheat flag leaves, wheat yield composition, and soil biological characteristics under O_3_ stress. Therefore, our aims were to (1) investigate the response of soil chemical properties, soil enzyme activities, and soil microbial communities to different N sources under O_3_ stress and (2) to evaluate the effects of different fertilizer management models on wheat physiological indices and yield and clarify the potential mechanisms of influence between above-ground plant physiology and subsurface biological processes.

## 2. Results

### 2.1. Effect of O_3_ and SCNF on Agronomic Parameters

**Figure 1:**
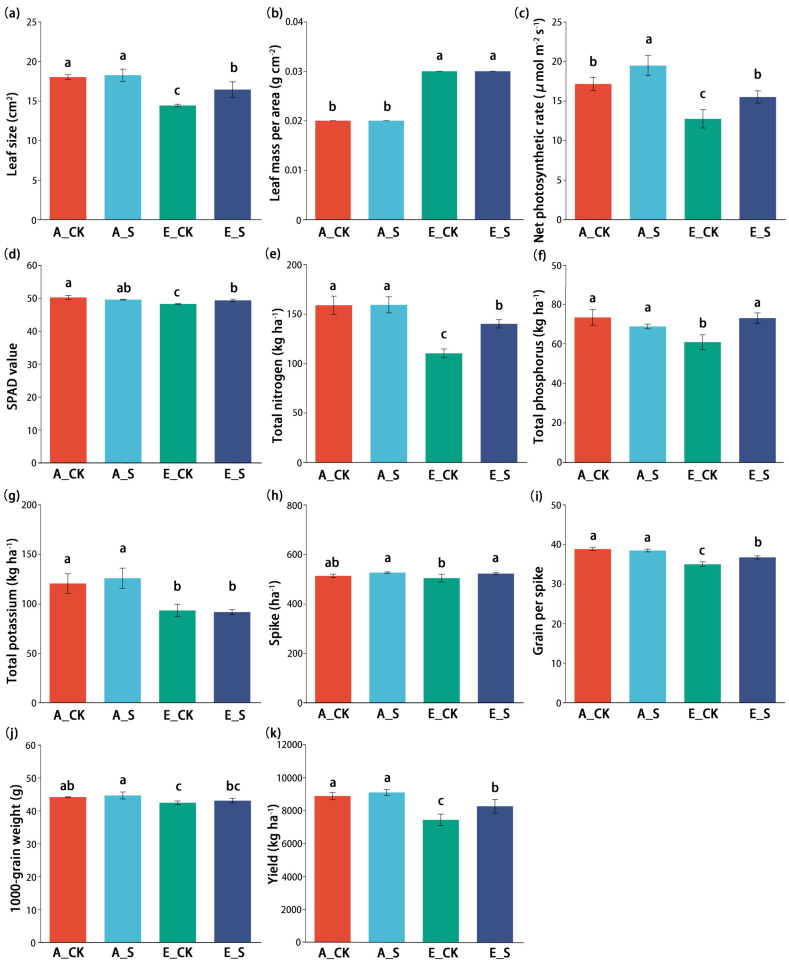
(**a**–**d**) Photosynthetic functions of wheat flag leaves in the flowering period (Z60), (**e**–**g**) nutrient accumulation of functional leaves, and (**h**–**k**) yield components at the maturity stage (Z92). A_CK, normal atmospheric environment + urea; A_S, normal atmospheric environment + sulfur-coated controlled release nitrogen fertilizer (SCNF); E_CK, ozone-free-air controlled enrichment (O_3_-FACE) + urea; E_S, O_3_-FACE + SCNF. Error bars mean the standard error and the different lowercase letters indicate significant differences between various treatments based on a one-way ANOVA followed by Duncan’s multiple-range tests (*p* < 0.05).

The photosynthetic functions of wheat flag leaves at the flowering period (Z60), accumulation of nutrient elements, and yield components at the maturity stage (Z92) were measured under different treatments (Figure 1). The results showed that leaf size (LS), net photosynthetic rate (*Pn*), and SPAD value decreased significantly (*p* < 0.05) under the O_3_-polluted conditions (E_CK and E_S); while leaf mass per area (LMA) was significantly increased. Compared with E_CK, the LS, *Pn*, and SPAD values of E_S were significantly improved (Figure 1a–d).

The premise of wheat yield is the photosynthetic capacity and nutrient accumulation of functional leaves. The accumulation of TN, TP, and TK (Figure 1e–g) at the maturity stage (Z92) was significantly positively correlated with yield, respectively (Spearman’s *r* value = 0.97, 0.58, 0.89, respectively). Under O_3_ stress, the nutrient accumulation and wheat yield components (Figure 1h–k) decreased significantly. However, the O_3_ stress was alleviated by the fertilizer management mode of SCNF replacing urea, which was reflected in the agronomic parameters of E_S being significantly higher than those of E_CK.

### 2.2. Effect of O_3_ and SCNF on Soil Chemical Properties

**Figure 2:**
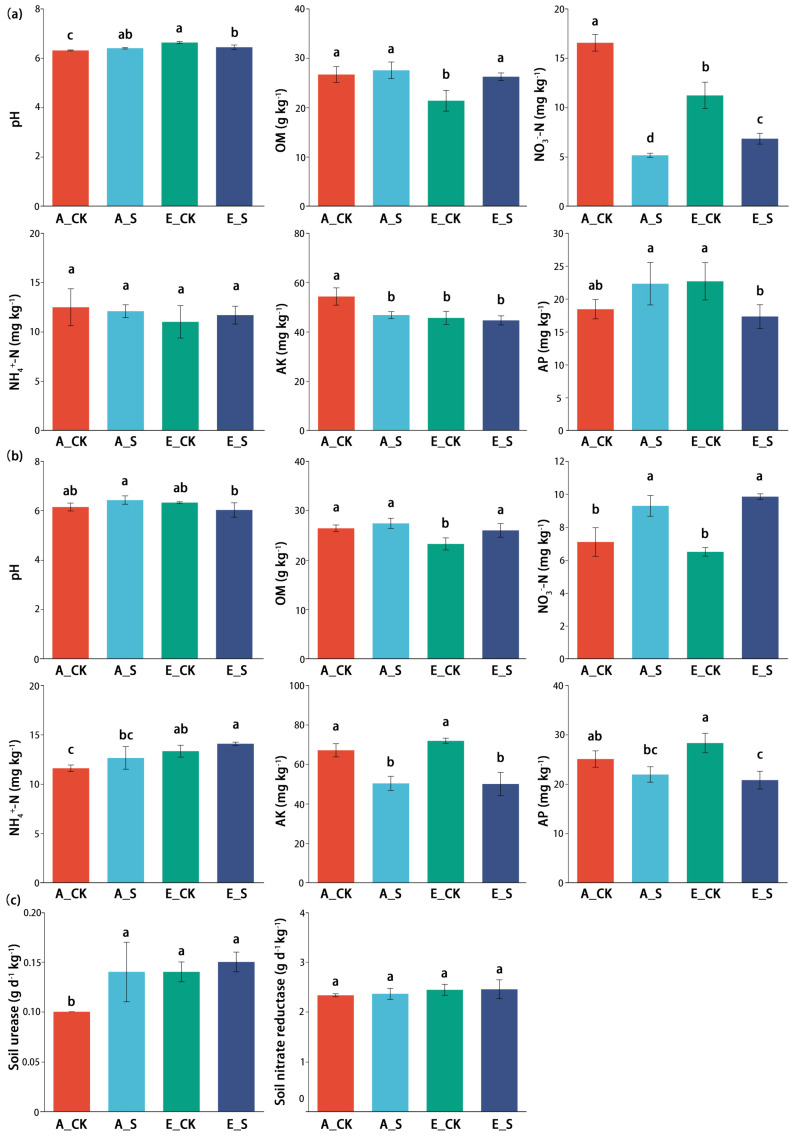
Soil chemical properties at the (**a**) flowering (Z60) and (**b**) mature period in different treatments and (**c**) activities of soil urease and soil nitrate reductase at the flowering period (Z60). OM, organic matter; NO_3_^−^–N, nitrate nitrogen; NH_4_^+^–N, ammonium nitrogen; AK, available potassium; AP, available phosphorus. Error bars mean the standard error and the different lowercase letters indicate significant differences between various treatments based on a one-way ANOVA followed by Duncan’s multiple-range tests (*p* < 0.05).

The soil chemical properties of each treatment at flowering (Z60) and maturity (Z92) stages are shown in Figure 2a,b. In these two growth stages, soil pH and content of OM were relatively stable, while the content of inorganic-N (NO_3_^−^–N and NH_4_^+^–N) fluctuated significantly. Both O_3_ and SCNF significantly promoted the activity of SUE (soil urease) at the wheat flowering period (Z60), but had no significant effect on the activity of SNR (soil nitrate reductase; Figure 2c).

Multiple variance analysis showed that the interaction of O_3_ and SCNF had a significant effect on the photosynthetic function of wheat flag leaves and soil chemical properties at the flowering period (Z60) and then on nutrient accumulation and yield at the mature period (Table 1).

### 2.3. Effect of O_3_ and SCNF on Microbial Diversity and Community Structure

#### 2.3.1. Quality Evaluation of Metagenomic Sequences

Nucleobase quality and distribution maps of the original sequences (Figure S1) showed that contents of base pair G–C and A–T were equal and remained stable throughout the entire sequencing process with horizontal lines. It is indicated that the construction quality of the metagenomic library was relatively high and the results were reliable. The genetic libraries of bacteria, fungi, and archaea were extracted and constructed for subsequent analysis.

#### 2.3.2. α Diversity Analysis of Soil Microbial Communities

High α diversity is a reflection of the health of microbial communities and the stability of soil ecosystem functions [36]. In this study, the commonly used α diversity metrics (Chao, Shannon, and Simpson indexes) were used to measure the richness and diversity of microbial communities in each treatment. The Chao index measures the richness of the number of species in the community; the Shannon and Simpson index reflects the diversity and stability of the community from the perspective of the status and role of dominant species in the community. In general, the Simpson index is negatively correlated with other α diversity indices.

In this study, it was observed that the response of the soil bacterial community to O_3_ was not reflected in α diversity. However, O_3_ had obvious stress on soil fungal and archaeal communities, reducing the Chao and Shannon index (Table 2). Replacing urea with SCNF significantly changed the diversity and stability of soil bacterial and archaeal communities, while the effect on α diversity of fungal community was not obvious.

#### 2.3.3. Structural Compositions and Differences in Soil Microbial Communities

**Figure 3:**
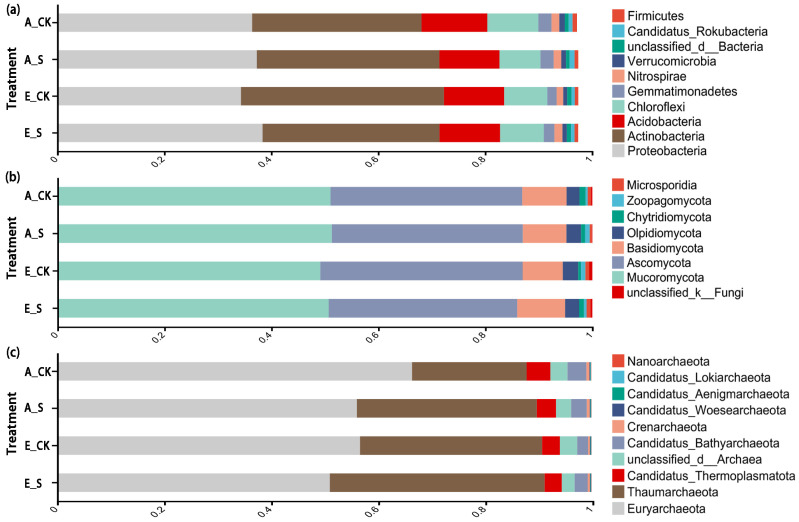
Influence of O_3_-FACE combined with SCNF on the relative abundance of soil (**a**) bacterial; (**b**) fungal; and (**c**) archaeal phyla. Only the phyla with RPKM ≥ 1% are presented in this figure.

The top 10 dominant phyla were selected for comparison (Figure 3). Each treatment had the same dominant strains, and the total relative abundance of the top five dominant phyla was more than 90%. However, the relative abundance of the same dominant strain in each treatment was different. The use of SCNF increased the relative abundance of dominant phyla in soil bacterial and fungal communities (Figure 3a,b). While the soil archaeal community showed a very different response (Figure 3c), the relative abundance of the top two archaea (*p_Euryarchaeota* and *p_Thaumarchaeota*) was significantly affected by O_3_ and SCNF.

#### 2.3.4. β Diversity Analysis of Soil Microbial Communities

**Figure 4:**
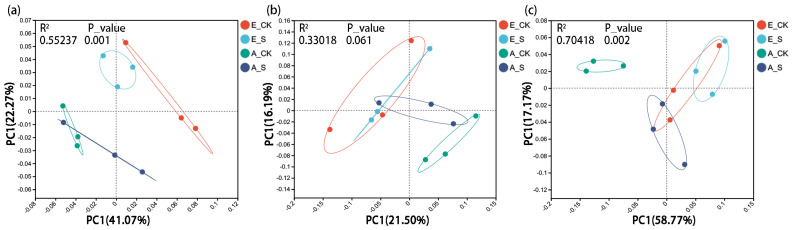
The principal component analysis (PCoA) and PERMANOVA at 99% level based on Bray-Curtis distance of soil (**a**) bacterial; (**b**) fungal; and (**c**) archaeal communities at the species level in the various treatments.

The principal co-ordinates analysis (PCoA) was carried out on the four treated soil microorganisms at the species level. The P_values of permutational multivariate analysis of variance (PERMANOVA) of bacterial and archaeal communities were less than 0.05, indicating that there were significant differences among the sample groups (Figure 4). The results display that the samples in different treatments represent obvious intra-group aggregation and inter-group dispersion.

It is worth noting that the PCoA results of the fungal community showed that the explanation of the PC1-axis (21.50%) and R^2^ value (0.33) were significantly lower than those of bacterial and archaeal communities, and the P_value (0.061) was greater than 0.05 (Figure 4b), indicating that the effects of O_3_ and SCNF on soil fungal community were much weaker than those on bacterial and archaeal communities. The explanation for the differences in soil fungal communities among the four treatments may not be the different atmospheric environments or nitrogen management models.

### 2.4. Interaction among Soil Microbial Communities, Soil Chemical Properties, and Agronomic Parameters

Many of the environmental factors commonly analyzed in the study related to the changes in microbial communities are autocorrelated. Therefore, before the interaction analysis of microorganisms and environmental factors, the variance inflation factor (VIF) is used to screen environmental factors to avoid the influence of autocorrelation environmental factors on the accuracy of subsequent analysis results [37]. The VIF values of the eight environmental factors (pH, OM, NO_3_^−^–N, NH_4_^+^–N, AK, AP, SUE, and SNR) selected in this experiment were all less than 10 (Table S1), indicating that the selection of environmental factors was scientific and there was no autocorrelation among them.

**Figure 5:**
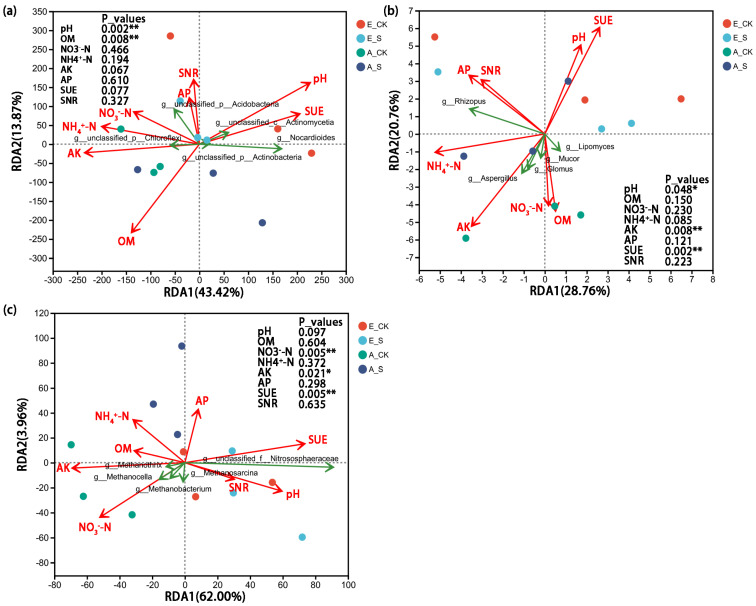
Redundancy analysis (RDA) of soil (**a**) bacterial; (**b**) fungal; and (**c**) archaeal genera with soil chemical properties. The soil biochemical properties were fitted to the ordination plots using a 999–permutation test (P_value). OM, soil organic matter; NO_3_^−^–N, nitrate nitrogen; NH_4_^+^–N, ammonium nitrogen; AP, available phosphorus; AK, available potassium; SUE, soil urease; SNR, soil nitrate reductase. Asterisks indicate significant differences at * *p* < 0.05 and ** *p* < 0.01.

Redundancy analysis (RDA) was used to quantify the effect of soil environmental factors on soil microbial communities at the genus level (Figure 5). The two environmental factors (pH and SUE) showed a positive correlation (the arrows were in the same direction) and their effects on the microbial communities showed opposite trends to four environmental factors (AK, OM, NO_3_^−^–N, and NH_4_^+^–N) (the arrows were in the opposite direction). Soil pH, SUE, and AK are all long arrows in Figure 5a–c, which can be considered as the three important environmental factors that have great impacts on soil microbial communities. The effect of soil NH_4_^+^–N on bacterial and fungal communities was stronger than that of NO_3_^−^–N; however, in the archaeal community, the opposite was true, and it was found that SCNF had a very significant effect on a high-abundance archaea genus (*g_unclassified_f_Nitrososphaeraceae*), which is directly involved in the N-cycling.

**Figure 6:**
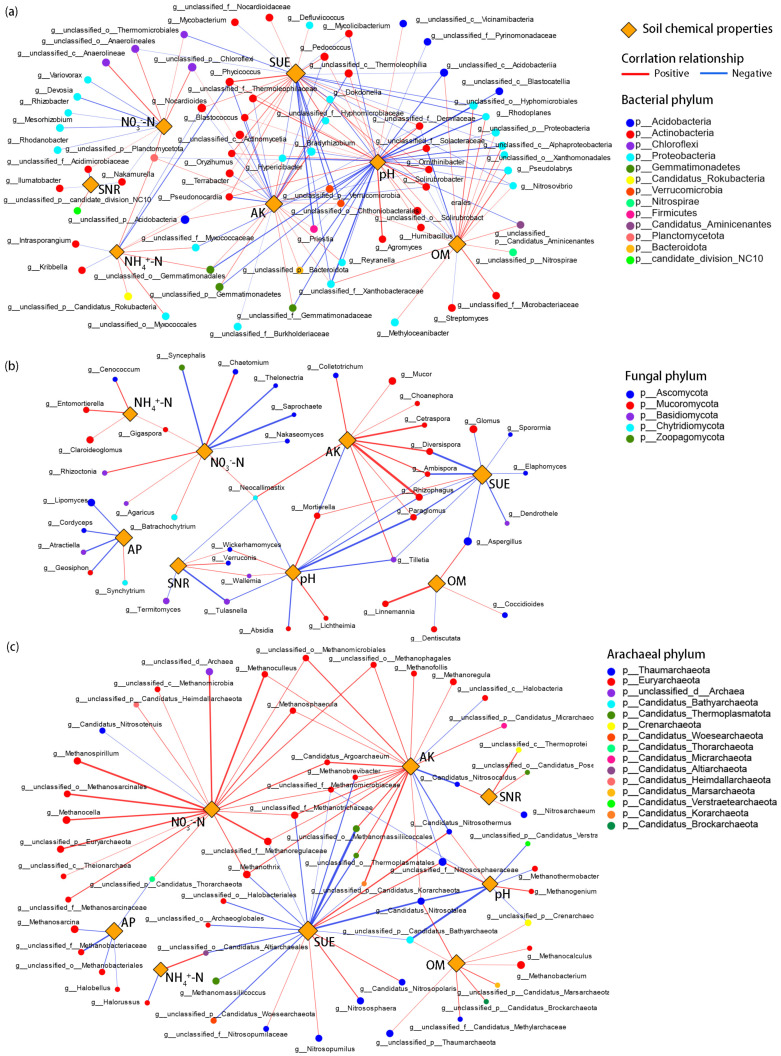
Correlation network between soil (**a**) bacterial; (**b**) fungal; and (**c**) archaeal genera and soil properties based on Gephi 0.9.2 software. Each network node represents a genus; its color and size correspond to the phylum to which it belongs and the relative abundance, respectively. The color and thickness of the network edge are expressed in the Spearman correlation and *r* value between the genus and the environmental factor, respectively.

Based on the Spearman correlation coefficient between soil microorganisms and soil chemical properties, the correlation networks of soil microbial genera–soil environmental factors were constructed to analyze the possible interactions. In addition to soil pH, SUE and AK, NO_3_–N, and OM were also important soil environmental factors affecting bacteria community structure at the genus level (Figure 6a). Many genera of *p_Actinobacteria* were positively correlated with soil pH and SUE but negatively correlated with AK and OM. There was maybe an inhibitory relationship between *p_Proteobacteria* and *p_Actinobacteria*, which showed that many of these genera tend to adapt to an environment of low SUE and pH and a high AK and OM environment. Many bacterial genera in *p_Chloroflexi* only showed significant positive correlations with the soil NO_3_^−^–N level and were not significantly affected by other soil environmental factors. The correlation networks between soil fungal and archaeal communities and environmental factors were clearer and simpler than those of soil bacteria. In soil fungal communities, *p_Ascomucota* and *p_Mucoromucota* showed strong responses to changes in soil environmental factors (Figure 6b). For the correlation network of archaeal genera, many genera of *p_Thaumarchaeota* and *p_Euryarchaeota* showed an obvious preference for an environment of high NO_3_^−^−N, AK, and OM, and low AP and SUE (Figure 6c).

**Figure 7:**
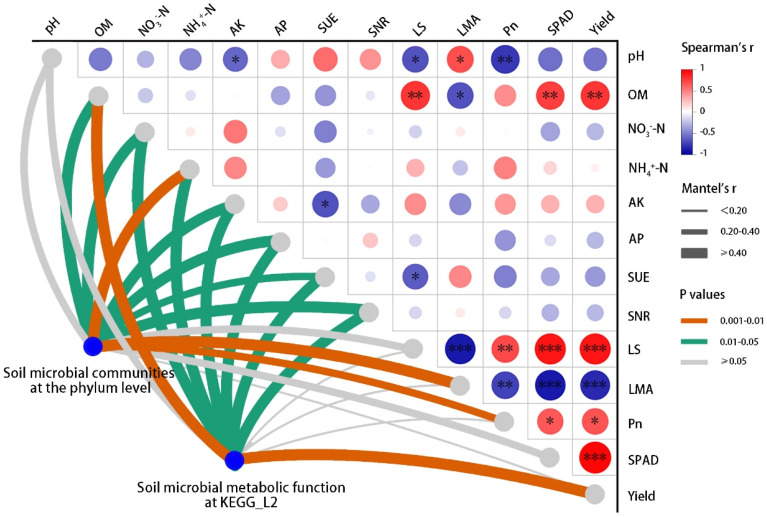
Pairwise comparisons of soil variables, agronomic parameters, and wheat yield. LS, leaf size; LMA, leaf mass per area; *Pn*, net photosynthetic rate. Mantel tests depict the association between soil microbial phyla and metabolic functions at the KEGG level 2 with environmental factors, respectively. The width of each edge matches Mantel’s *r* statistic, and the color represents Mantel’s P value (Table S2). Asterisks indicate significant differences at * *p* < 0.05, ** *p* < 0.01 and *** *p* < 0.001.

O_3_ and SCNF jointly affect the wheat yield by affecting the above-underground ecological processes. Spearman correlation analysis and mantel test were used to examine the possible interaction effects between environmental factors and soil microbial communities on the agronomic parameters of wheat in this study (Figure 7). Among these soil environmental factors, pH and OM showed opposite influencing mechanisms on photosynthetic function (LS, LMA, *Pn*, and SPAD value) of flag leaves during the flowering period (Z60) and wheat yield. Unsurprisingly, the photosynthetic function of flag leaves showed extremely strong correlations with yield.

From another aspect, the results of mantel tests verify the reliability of RDA analysis results (Table S2). The community structure of soil microbial phyla and metabolic function at KEGG level 2 were strongly correlated with soil chemical properties and wheat agronomic parameters, respectively. The difference was especially shown in the results of mantel tests on wheat yield. Although the soil microbial community at the flowering period (Z60) did not show a significant association with yield, the metabolic functions of soil microorganisms did show responses.

### 2.5. Comparison of Abundance Differences in N-Cycling Genes

At the same N supply level in all treatments, we believe that in addition to the effect of O_3_, the difference in the release rate of inorganic-N by urea and SCNF would also affect the N-cycling process of soil microorganisms, and the significant difference in SUE was evidence (Figure 2c). By comparing the sequences of metagenomic sequencing with the KEGG library, the metabolic pathways related to the N-cycling (*ko00910*) were screened for significant difference tests (Figure S2). The results showed that each process of soil N-cycling was affected by environmental factors, and the abundances of related functional genes were significantly different among different treatments.

**Figure 8:**
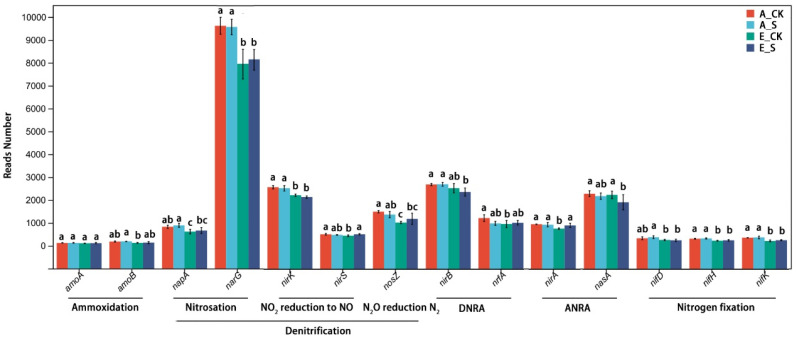
Variance analysis of 14 key functional genes involved in N-cycling. DNRA, dissimilatory nitrate reduction ammonia; ANRA, assimilatory nitrate reduction ammonia. Different lowercase letters mean significant difference based on a one-way ANOVA followed by Duncan’s multiple-range tests (*p* < 0.05).

The abundance of 14 key functional genes involved in the important processes of N-cycling was selected for variance analysis, including ammoxidation (*amoA*, *amoB*), denitrification (nitrosation (*napA*, *narG*), NO_2_ reduction to NO (*nirK*, *nirS*), and N_2_O reduction (*nosZ*)), assimilatory (*nirB*, *nrfA*) and dissimilatory (*nirA*, *nasA*) nitrate reduction ammonia, and nitrogen fixation (*nifD*, *nifH*, *nifK*) (Figure 8) [22]. Under the O_3_-FACE condition, except for the *amoA* gene, the abundances of the other 13 functional genes were significantly reduced, indicating that the activities and metabolic functions of soil microorganisms were significantly inhibited. In addition, under a normal atmospheric environment, the responses of N-cycling-related genes to different fertilizer management modes were not significant. However, under the O_3_ stress, SCNF could significantly increase the abundance of these genes, which once again proved the mitigation effects of SCNF on O_3_ inhibition.

## 3. Discussion

### 3.1. Effects of O_3_ and SCNF on Photosynthetic Parameters of Wheat Flag Leaves

O_3_-induced changes in parameters of leaf photosynthetic function are important indicators for assessing crop adaptive traits and can be widely used in conventional field surveys [38]. In this study, the O_3_ stress environment had obvious damage to the photosynthetic function of wheat flag leaves at the flowering period (Z60), especially for LS and LMA (Table 1). LS is an indicator to measure the material transmission capacity between leaves and the environment; LMA is negatively correlated with photosynthetic rate, but positively correlated with leaf longevity [39]. The changes in LS and LMA in this study indicated that wheat sacrificed the photosynthetic rate self-regulation strategy to reduce ozone uptake amount and prolong leaf life under ozone stress. The changes in LS and LMA in this study indicated the self-regulation strategy of wheat sacrifice to the photosynthetic rate in order to reduce ozone absorption and prolong leaf life under O_3_ stress. Similar results were reported by Feng et al. [39] and Li et al. [40]: as an important leaf attribute index, LMA indicates the risk of a stressful environment and the improvement in LMA is a “slow return” strategy for leaves to improve resource utilization in stress environments.

The photosynthetic parameters of flag leaves at the flowering stage (Z60) showed very strong correlations with the wheat yield at the maturity stage (Z92; Figure 7). Multivariate variance analysis showed that different nitrogen fertilizer treatments had no significant effect on LMA and SPAD value (Table 1), indicating that the nitrogen fertilizer management mode of SCNF instead of urea could not delay the O_3_-induced leaf senescence of winter wheat. However, SCNF could increase nutrient accumulation amount by increasing LS and *Pn*, which was the physiological basis for alleviating the inhibitory effect of O_3_ on wheat yield formation.

### 3.2. Regulation of O_3_ and SCNF on Nutrient Accumulation and Wheat Yield

The nitrogen fertilizer management method used in A_CK and E_CK treatments is one of the high-yield practices vigorously promoted in long-term agricultural production in Jiangsu Province. In this experiment, under a normal atmospheric environment, compared with the four times application of urea, the twice-split application of SCNF can reduce the management cost and provide a higher yield (Figure 1h–k). At the same level of nitrogen supply, we believe that the fundamental difference between the urea and SCNF treatments is essentially related to the level and timing of the soil’s inorganic-N supply [29].

But surprisingly, SCNF showed effectiveness in alleviating O_3_ stress. Compared with that of E_CK treatment, the accumulation amount of nutrients and components of wheat yield (Figure 1) of the E_S treatment were significantly increased. The number of spikes per hectare is one of the key factors of wheat yield components. Related studies have reported that O_3_ has a great side effect on the formation of productive ears from tillers during the reproductive growth phase [41]. Under the condition of unifying the planting density of basic seedlings and the same field management, O_3_ significantly inhibited the formation of productive ears [42], while SCNF could increase the tiller number and ear-bearing tiller rate of wheat and greatly alleviate the decrease in wheat yield caused by O_3_ (Figure 1).

### 3.3. Effects of O_3_ and SCNF on Soil Chemistry Properties

Through conventional statistical analysis, it was found that the soil pH and OM content were relatively stable, while the inorganic-N content fluctuated significantly during the flowering (Z60) and maturity (Z92) stages of winter wheat (Figure 2). Multivariate variance analysis showed that soil pH and OM responded strongly (*p* < 0.01) to O_3_ (Table 1). Similar conclusions have reported that O_3_, as a strong oxide, can increase soil pH, accelerate the oxidation and decomposition of organic matter, and reduce the soil soluble organic carbon by increasing the soil redox potential [43]. More importantly, O_3_-induced soil pH increase is one of the most important factors affecting the denitrification process and N_2_O production [44]. However, the effect mechanism of SCNF on N-cycling needs to be further discussed in combination with changes in the abundance of related genes.

Multivariate variance analysis also showed that the changes in soil inorganic-N did not show interaction with O_3_, but were significantly affected by SCNF (Table 1). Consistent with previous studies, although the use of SCNF could not rapidly increase the content of soil NO_3_^−^–N in the short term compared with urea, it provided a long-term N supply and increased the retained soil inorganic-N by delaying the N release rate (Figure 2) [30].

### 3.4. Responses of the Soil Microbial Community Structure to O_3_ and SCNF

Soil microbial communities are very sensitive to the change in soil environment. The soil’s physical and chemical properties will directly affect the nutrient supply capacity and living environment of soil microorganisms [45]. Studies have mentioned that O_3_ exposure and different fertilizer management models can affect the structure and diversity of soil bacterial and fungal communities [7]. Similar results were also found in this study. Although the compositions and dominant strains of soil microorganisms did not change, different treatments had significant effects on the α (Table 2) and β (Figure 4) diversity. In addition, PCoA results showed that the β diversity of soil microorganisms was more sensitive to the effect of O_3_ than SCNF (Figure 4).

*p_Proteobacteria*, *p_Actinobacteria*, and *p_Acidobacteria* were the three most abundant bacterial phyla in the soil, accounting for more than 80% of the total relative abundance, which was consistent with the results of previous studies [46]. *p_Actinobacteria* is a microbial community with a strong stress tolerance in the soil bacterial community [47,48]. In this study, the relative abundance of *p_Actinobacteria* in E_CK was significantly higher than that in the other three treatments (Figure 3), indicating that *p_Actinobacteria* was obviously more adapted to the stress environment of elevated O_3_ than other soil microbial phyla. Moreover, it is also proven that SCNF can adapt microorganisms to a high O_3_ environment by regulating the soil environment [45].

Many crop-related studies have discussed the effects of different field management methods on soil fungal communities and explored the interaction between crop production and soil fungi [7,49]. In this study, the PCoA results showed that the PC1-axis interpretation of fungi was much lower than that of bacteria and archaea (Figure 4), indicating that soil bacteria and archaea were more sensitive to changes in environmental factors than fungi. Relevant studies have proposed an explanation that the main reason affecting the soil fungal community structure is soil-borne fungal diseases rather than different fertilization systems or rotation patterns [50].

### 3.5. Interactions between Environmental Factors and Soil Microorganisms

The results of RDA showed that there were obvious symbiotic or inhibitory relationships between some genera in the bacteria (*p_Actinobacteria*, *p_Actinomycetia*, *p_Chloroflexi*, etc.), fungi ((*p_Mucoromycota*, *p_Ascomycota*, etc.), and archaea phyla (*p_Euryarchaeota*, *p_Thaumarchaeota*, etc.) (Figure 5). The correlation network confirmed that soil pH, AK, SUE, and NO_3_^−^–N are the key soil environmental factors that changed the soil microbial community structure in this study (Figure 6). Aller and Kemp [51] also concluded that pH and C/N were the key soil factors affecting the archaeal community structure. Unsurprisingly, the results of mantel tests further verified the correlations between key soil environmental factors and microbial genera.

It is worth noting that SCNF could affect the relative abundance of soil N-cycling-related microorganisms by changing the nitrogen release rate. Many microbial genera were identified to be significantly positively (*p_Chloroflexi*, *p_Euryarchaeota*, etc.) or negatively (*p_Proteobacteria*, *p_Ascomycota*, etc.) related to soil NO_3_^−^–N, respectively (Figure 6), suggesting that these genera may play an important role in soil N-cycling or have a strong response to changes in soil NO_3_^−^–N content [30]. SCNF had a significant effect on *p_Thaumarchaeota*, a high-abundance ammonia-oxidizing archaea (AOA) directly involved in N-cycling, in which multiple biomarkers (*f_Nitrosopumilaceae*, *f_Nitrososphaeraceae*, etc.) with significantly increased relative abundance were identified, suggesting a high impact of SCNF on the N-cycling process [52].

### 3.6. Responses of Soil Microbial Nitrogen Metabolism to O_3_ and SCNF

The response of soil microorganisms to environmental stress usually reflects the community structure and biological processes and the response of biological processes often occurs earlier than the change in community structure [15]. Relevant studies have suggested that O_3_ will destroy the N-cycling process driven by soil microbial activity, which may inhibit many important processes of N transformation such as nitrogen fixation, nitrification, and denitrification and have a negative impact on N_2_O emissions from farmland [37]. Similar results were obtained in this study. The genes controlling related enzymes in the nitrogen metabolism pathway (*ko00910*) were significantly affected by O_3_ (Figure S2) and the abundances of functional genes related to N-cycling were significantly reduced (Figure 8). Moreover, SCNF significantly alleviated the inhibitory effects of O_3_ on these related genes.

It is worth noting that under the same supply level of soil inorganic nitrogen, the nitrogen fertilizer management mode of SCNF instead of urea could significantly increase the inorganic nitrogen retained in the soil at the maturity stage (Z92) of winter wheat (Figure 2). We believed that SCNF could increase the abundance of genes related to nitrogen fixation (*nifD*) and ammoxidation (*amoB*) processes to retain more NO_3_^−^–N. In addition, it may be possible for N_2_O emissions to be indirectly reduced by inhibiting the abundance of genes related to NO_2_ reduction to NO (*nirK, nirS*) and N_2_O reduction (*nosZ*) processes.

### 3.7. Applicability and Limitations

From the perspective of wheat production and soil biological characteristics, our study confirmed that the use of SCNF instead of urea can promote the photosynthetic function of flag leaves, improve the soil environment, stabilize the microbial community, and increase the yield of winter wheat by providing long-term N supply. This will become an effective mitigation measure to cope with the situation of elevated ambient O_3_. However, these conclusions will require years of experimental verification. These results will be presented in a future series of reports.

Additionally, it is undeniable that there are still some shortcomings in this study. Some studies have proposed that the main climatic factors affecting the rice-wheat rotation ecosystem in Jiangsu Province are not only the elevated ambient O_3_, but also the continuous increase in atmospheric CO_2_ and land surface temperature [53]. Miao et al. [54] suggested that CO_2_ and temperature even showed an interactive superposition effect on farmland ecosystems. Therefore, the study of the impact of climate change on farmland ecosystems urgently needs to consider the combined effects of multiple climate factors on the entire structure and function of farmland ecosystems.

## 4. Materials and Methods

### 4.1. Experimental Site

The experimental site is located in the research and demonstration base of green agriculture (119°43′ E, 32°25′ N) in Jiangdu District, Yangzhou City, China. The local implementation of a long-term rice-wheat rotation system is one of the typical farmland ecosystems in China [42].

**Figure 9:**
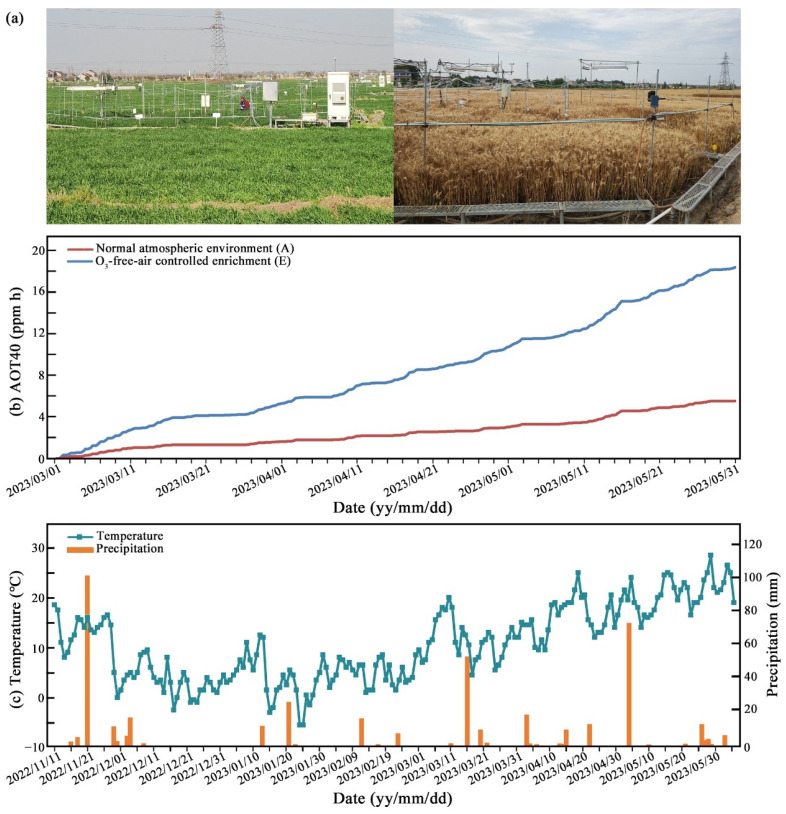
(**a**) The ozone-free-air controlled enrichment (O_3_-FACE) simulation platform. (**b**) AOT40 value during the treatment phase of elevated ozone. (**c**) The average temperature and total precipitation at the experimental field during the wheat growing season in 2022–2023.

The ozone-free-air controlled enrichment (O_3_-FACE) simulation platform was built in 2019 and has been pre-tested for many years to consistently simulate elevated ambient O_3_ in field environments. Each built-up FACE zone is an octagonal structure with a diameter of 12 m and an effective area of about 120 m^2^ (Figure 9a). In order to avoid the influence of gas diffusion on the control field of the normal atmospheric environment, the distance between each FACE zone is more than 90 m.

The experiment used the WJ-H-Y5 O_3_ generator (Wanjie Ozone Electromechanical Equipment Factory, Nanjing, China) to produce O_3_ by electrolysis of oxygen in the atmosphere. The O_3_ release tubes equipped in each FACE zone were consistent in model and layout and were kept at a height of 30–50 cm from the ground during the wheat growing season. O_3_ was ejected from these tubes above the wheat canopy and diffused freely into the FACE zones to achieve a stable concentration increase. The valves and O_3_ concentration sensors (accuracy: 1 nL L^−1^) were set in the tubes to realize the automatic control of O_3_ concentration in the O_3_-FACE zone. In addition, the experimental site was also equipped with a small meteorological station, using O_3_ detectors (Model S200, aeroQUAL Co., Auckland, New Zealand) to monitor the O_3_ concentration in each FACE zone, to provide data for the control system of simulating different atmospheric environments.

From 1 March 2023 to the wheat harvest (3 June 2023), O_3_ gas was sprayed daily from 8 a.m. to 5 p.m. and the real-time monitoring data of the accumulated hourly ozone concentrations over a cut-off threshold of 40 ppb (AOT40) are shown in Figure 9b. During the wheat-growing season from 2022 to 2023, the average temperature and total precipitation in the experimental site were 10.6 °C and 412.3 mm, respectively (Figure 9c).

### 4.2. Materials and Treatments

The experimental material was Nongmai-88, which is one of the main and superior cultivated varieties of winter wheat in Jiangsu Province, China. Nongmai-88 ranks first among the strong gluten varieties in China and its grain quality reaches the first-class strong gluten standard of the national standard GB/T 17892-1999 [55]. Nongmai-88 is a medium–low growth and medium–early maturity wheat variety, which has shown advantages of high and stable yield in regional experiments and production experiments in Jiangsu Province. The whole growth period was about 208 days, and the planting density was 225 × 10^4^ per hectare after removing excess seedlings manually at the three-leaf stage. The growth stage of wheat corresponds to the Zodaks scale.

The experimental soil was silt loam, and the basic soil productivity of the topsoil (0–20 cm) was evaluated before sowing (Table 3).

A two-factor randomized block design was used to explore the interaction effects between O_3_ and SCNF on the physiology of winter wheat and soil biological characteristics. A treatment structure comprising two main-plot factors (normal atmospheric environment (A) and elevated ambient O_3_ concentration (E)) and two sub-plot factors (urea (CK) and SCNF (S)) arranged in a split-plot design was used for this study. (1) A_CK, normal atmospheric environment + urea; (2) A_S, normal atmospheric environment + SCNF; (3) E_CK, elevated ambient O_3_ concentration + urea; and (4) E_S, elevated ambient O_3_ concentration + SCNF (Table S3). Each treatment was repeated three times.

According to the optimal fertilizer dosage for local wheat cultivation in the wheat industry development report of Jiangsu Province (http://www.jsnjy.net.cn/newsDetail.html?newId=3cf5ac14-869a-4af9-942c-861b6ec8b180, accessed on 10 September 2022), the fertilizer application amount of this experiment was determined to be 225–100–120 kg ha^−1^ (N–P_2_O_5_–K_2_O). The N fertilizers included in this study were SCNF (37% N) and common urea (46% N). The nutrient release longevity of SCNF was 3–4 months, and the sulfur coating begins to weather after about 6 months and is broken down by microorganisms to provide sulfur nutrients to crops after 10 months (purchased from Hanfeng slow-release fertilizer Co., Ltd., Taizhou, China). Urea, superphosphate (12% P_2_O_5_), and potassium chloride (60% K_2_O) were purchased from the local fertilizer distributor.

In treatments A_CK and E_CK, urea was used as the nitrogen fertilizer, and the application proportion of basal fertilizer: tillering fertilizer: jointing fertilizer: panicle fertilizer was 5:1:2:2. This method can achieve better coordination between quality and yield, has great economic benefits, and is one of the high-yield practices vigorously promoted in long-term agricultural production in Jiangsu Province [30]. In treatments A_S and E_S, SCNF was used instead of urea, with 60% applied as basal fertilizer and 40% topdressing at the re-greening stage (Z30). There was no additional irrigation except for rainwater, and field management followed a local high-yield cultivation management program. The phosphate and potassium fertilizers in each treatment were all applied as base fertilizers before sowing (Z00).

### 4.3. Plant Sampling and Analysis

The net photosynthetic rate (*Pn*) of wheat flag leaf was measured by the Li-6400 portable photosynthesis system (Li COR, Lincoln, NE, USA) under natural light from nine to eleven a.m. every 7 days in sunny weather [56]. The photosynthetic light-response curves were created using ten light intensity gradients at 1800, 1500, 1200, 900, 600, 300, 100, 50, 20, and 0 μmol m^–2^ s^–1^. Each treatment was repeated three times.

Twenty flag leaves were randomly selected for each treatment to determine the relative content of chlorophyll using a SPAD-502 Plus chlorophyll analyzer made by Minolta, Japan [57].

Plant samples were collected at the flowering (Z60) and maturity (Z92) stages, and the dry weight, nutrient content, and accumulation (total nitrogen, TN; total phosphorus, TP; total potassium, TK) of wheat organs (stem and sheath, leaf, grain, ear axis and glume) were measured after drying. The contents of TN, TP, and TK were determined by the indophenol blue method, molybdenum antimony colorimetric method, and flame photometry, respectively [58]. Ten representative rows were selected in each field, the wheat yield components were measured at the maturity stage (Z92), and the 1000-grain weight and yield were calculated at 13% moisture content.

### 4.4. Soil Sampling and Assessment of Chemical Properties

At the flowering (Z60; 19 April 2023) and mature period (Z92; 3 June 2023), topsoil (0–20 cm) samples were collected by a five-point sampling method [59]. After visible plant roots and organic residues were removed, fresh soil was taken by the quartering method, and the enzyme activities (soil urease, SUE; soil nitrate reductase, SNR) of soil samples were determined according to the instructions of the detection kits (Keming Biotechnology Co., Ltd., Suzhou, China). The remaining soil samples were dried and the soil chemical properties were determined. The soil pH was measured by the glass electrode method. A continuous flow injection analyzer (Model AA3–A001–02E, Bran–Luebbe, Norderstedt, Germany) was used to determine the contents of nitrate nitrogen (NO_3_^−^–N) and ammonium nitrogen (NH_4_^+^–N) in soil [60]. The potassium dichromate volumetric method, flame photometric method, and molybdenum antimony colorimetric method were adopted to measure the contents of soil organic matter (OM), available potassium (AK), and available phosphorus (AP), respectively [58].

### 4.5. Soil Metagenomic DNA Extraction, Sequencing and Data Analysis

Part of the mixed fresh soil samples were sub-packed in a sterilized 15 mL centrifuge tube and stored in liquid nitrogen [61]. After about 3 h, they were transported to the laboratory (Major Bio-Pharm Technology, Co., Ltd., Shanghai, China) for soil microbial metagenomic sequencing. After DNA extraction and sequencing, data quality control, and splicing assembly, non-redundant gene sets were constructed and the species annotation information and corresponding functional information were obtained by comparing with the NR and KEGG database by DIAMOND [62,63,64].

### 4.6. Statistical Analyses and Bioinformatics

The data of plant agronomic parameters and soil chemical properties were analyzed and processed with the SPSS 20.0 software (IBM Corp, Armonk, NY, USA), and the related images were drawn in Origin 8.0 (Origin Lab Corporation, Northampton, MA, USA). The significantly different means were separated using variance analysis (one-way ANOVA) followed by Duncan’s multiple-range tests at a 5% level of probability [65].

The microbial abundance was calculated by the reads per kilobase million (RPKM) method [66]. Bioinformatics data were analyzed using the R statistical software package in RStudio version 0.99.446 (Rstudio, Inc., Boston, MA, USA, 2015).

## 5. Conclusions

In conclusion, the results of the field experiment showed that SCNF could promote the photosynthetic function of wheat flag leaves and improve the soil environment by providing long-term nitrogen supply and demonstrated the obvious potential of SCNF to alleviate O_3_ stress on winter wheat production. O_3_ had significant inhibitory effects on the physiology and production capacity of winter wheat, the stability of soil microbial communities, and the abundance of N-related metabolic functional genes; soil microbial metabolic functions responded earlier than the community structure of soil microorganisms. Although SCNF could not delay the O_3_-induced leaf senescence of winter wheat, it could effectively alleviate the damage to the photosynthetic function of wheat flag leaves, increase the leaf size to enhance the accumulation of dry matter and nutrients, lay the foundation for yield, and offset some of the effects on the wheat yield. In addition, SCNF significantly alleviated the inhibitory effect of O_3_ on functional genes related to N-cycling, which means that SCNF has great potential in regulating the soil N transformation process and increasing soil N retention. The results showed that the replacement of urea by SCNF would be an effective field nitrogen management strategy with both agronomic and ecological benefits under the situation of a continuous increase in ambient O_3_ in the future.

## Figures and Tables

**Table 1:** Multivariate variance analysis of the effects of O_3_ combined with SCNF on wheat agronomic parameters and soil properties.

			O_3_	SCNF	O_3_ × SCNF
Flowering period (Z60)	Leaf photosynthetic function	LS	**	*	NS
		LMA	**	NS	NS
		*Pn*	*	*	NS
		SPAD value	NS	NS	**
	Soil enzyme activity	SUE	*	*	NS
		SNR	NS	NS	NS
	Soil chemistry property	pH	*	NS	**
		OM	NS	*	NS
		NO_3_^−^–N	*	**	**
		NH_4_^+^–N	NS	NS	NS
		AK	*	*	NS
		AP	NS	NS	**
Mature period (Z92)	Yield structure	Spike	NS	*	NS
		Grain per spike	**	*	*
		TGW	*	*	NS
		Yield	*	**	NS
	Plant nutrient accumulation	TN	**	**	*
		TP	NS	*	**
		TK	**	NS	NS
	Soil chemistry property	pH	**	**	*
		OM	*	NS	NS
		NO_3_^−^–N	NS	**	NS
		NH_4_^+^–N	NS	NS	NS
		AK	NS	**	NS
		AP	NS	**	*

Note: O_3_, ozone-free-air controlled enrichment; SCNF, sulfur-coated controlled-release nitrogen fertilizer. LS, leaf size; LMA, leaf mass per area; *Pn*, net photosynthetic rate; SUE, soil urease; SNR, soil nitrate reductase; OM, organic matter; NO_3_^−^–N, nitrate nitrogen; NH_4_^+^–N, ammonium nitrogen; AK, available potassium; AP, available phosphorus; TGW, 1000-grain weight; TN, total nitrogen; TP, total phosphorus; TK, total potassium. NS, no significance; asterisk mark denotes the significance level: ** *p* < 0.01 and * *p* < 0.05.

**Table 2:** *α* diversity indices of microbial communities at the species level.

	Treatment	Chao Index	Shannon Index	Simpson Index
Bacteria	A_CK	21,435 ± 50 a	5.70 ± 0.01 b	0.016 ± 0.000 ab
	A_S	21,073 ± 84 a	5.79 ± 0.01 a	0.015 ± 0.000 b
	E_CK	20,507 ± 217 a	5.71 ± 0.01 b	0.017 ± 0.001 a
	E_S	20,794 ± 199 a	5.74 ± 0.00 ab	0.016 ± 0.000 ab
Fungi	A_CK	157.00 ± 2.33 a	3.60 ± 0.01 a	0.061 ± 0.003 a
	A_S	148.00 ± 1.86 a	3.54 ± 0.02 ab	0.068 ± 0.001 a
	E_CK	134.00 ± 2.91 b	3.48 ± 0.01 b	0.069 ± 0.002 a
	E_S	133.33 ± 1.07 b	3.53 ± 0.02 ab	0.066 ± 0.002 a
Archaea	A_CK	629.33 ±2.50 a	3.83 ± 0.01 a	0.004 ± 0.001 c
	A_S	602.00 ± 4.06 ab	3.69 ± 0.01 b	0.060 ± 0.002 bc
	E_CK	587.67 ± 7.56 b	3.61 ± 0.02 bc	0.072 ± 0.003 ab
	E_S	594.67 ± 8.70 ab	3.54 ± 0.03 c	0.086 ± 0.004 a

Note: Means are followed by ±s.e.m. Different lowercase letters mean significant difference based on a one-way ANOVA followed by Duncan’s multiple-range tests (*p* < 0.05).

**Table 3:** Primary properties of topsoil (0–20 cm) at the test field.

pH	Organic Matter (g kg^−1^)	Nitrate Nitrogen (mg kg^−1^)	Ammonium Nitrogen (mg kg^−1^)	Available Phosphorus (mg kg^−1^)	Available Potassium (mg kg^−1^)
6.7	30.07	43.26	2.26	17.11	60.38

## Data Availability

The data files (reads in FASTQ format) were deposited at the NCBI SRA database under the BioProject, accession No. PRJNA999761 (https://www.ncbi.nlm.nih.gov/sra/PRJNA999761, accessed on 29 July 2023).

## Supplementary Materials

The following supporting information can be downloaded at https://www.mdpi.com/article/10.3390/ijms25179381/s1.
