# Supplementary material for: Regulating Leaf Photosynthesis and Soil Microorganisms through Controlled-Release Nitrogen Fertilizer Can Effectively Alleviate the Stress of Elevated Ambient Ozone on Winter Wheat
Source: Int J Mol Sci. 2024 Aug 29;25(17):9381. doi: 10.3390/ijms25179381 (PMC11394819; doi:10.3390/ijms25179381)
Supplement: Supplementary file 1 [file ijms-25-09381-s001.zip › ijms-3176480-supplementary.pdf]

Supplementary Materials

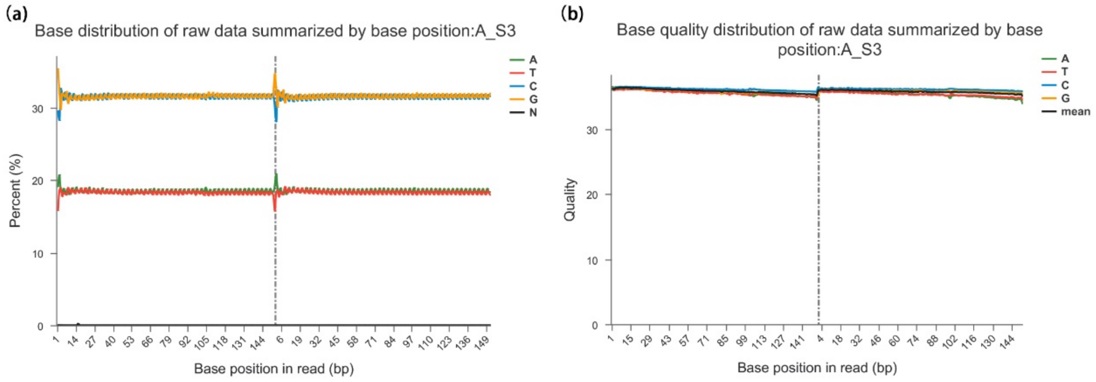

Figure S1. (a) Base distribution of raw data summarized by base position: A_S3. The abscissa is the base position coordinate of the reads, and the ordinate is the base quality of the reads. In this Fig., the range specified by the vertical "I" is the distribution interval of the base quality of all reads, the vertical square is the interquartile value range of quality, and the thick line is the median of the quality value. The left side of the dotted line shows the base mass of read1, and the right side shows the base mass of read2. (b) Base quality distribution of raw data summarized by base position: A_S3. Show whether there is A-T, G-C separation and whether the library is uniform. The abscissa is the base coordinate of the reads, and the ordinate is the percentage of bases A, C, G, and T of all reads at the sequencing position (such as the first sequencing base). Different bases are represented by different colors.

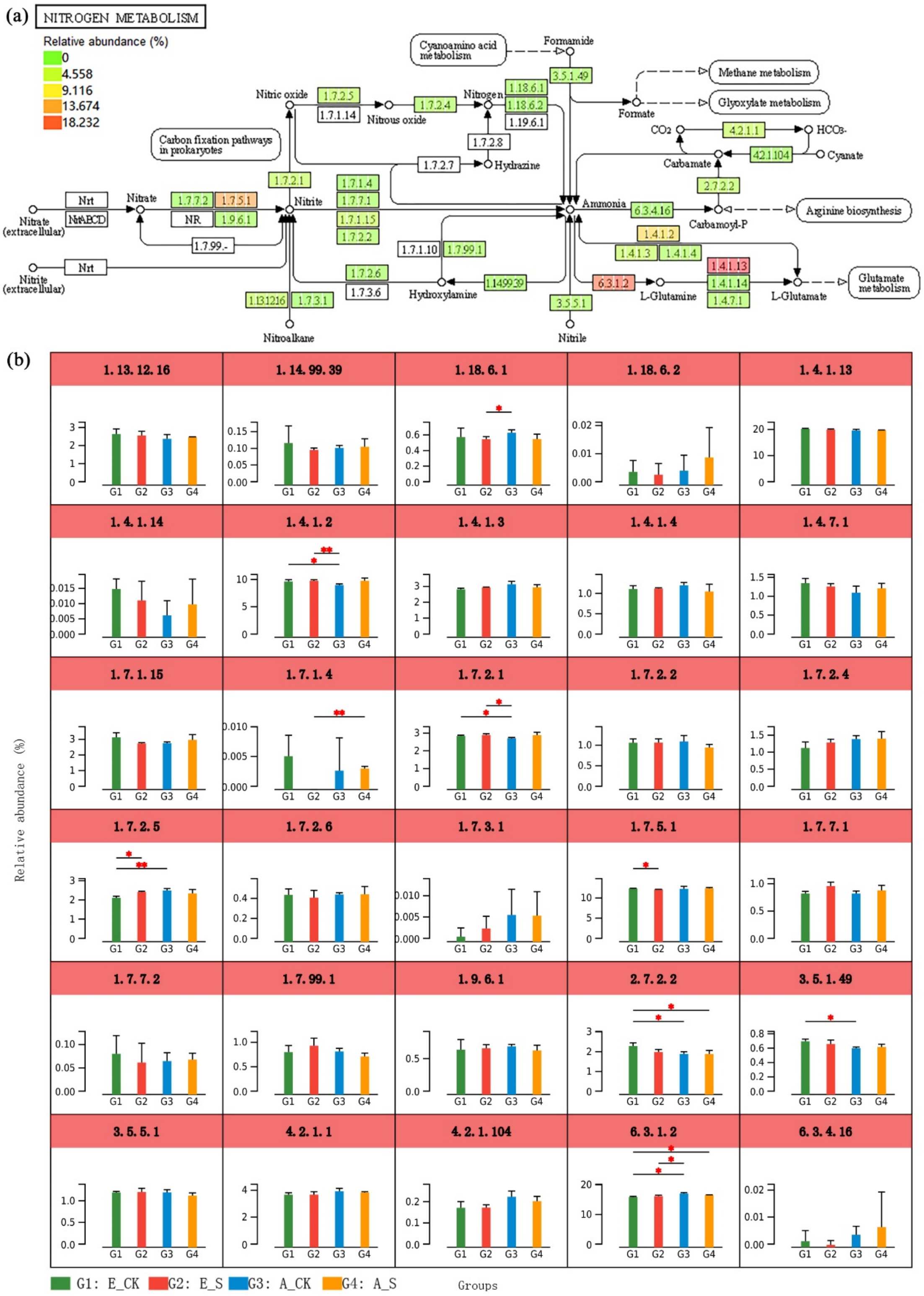

Figure S2. (a) The abundance of nitrogen-related genes in nitrogen metabolic pathway (*ko00910*). The numbers in the box are enzyme IDs. (b) The significance tests for abundance of nitrogen-related enzymes. * indicates significant difference between two treatments based t-test (*, P < 0.05; **, P < 0.01).

Table S1. VIF value of eight soil environmental factors.

Soil chemical properties	VIF Value
pH	5.84
OM	5.56
$NO3^{-}$–N	5.49
$NH4^{+}$–N	3.51
AK	8.8
AP	1.72
SUE	6.73
SNR	1.91

Note: SUE, soil urease; SNR, soil nitrate reductase.

Table S2. Mental test of soil properties with microbial communities at the phylum level and metabolic function at KEGG_L2.

Properties		Microbial phyla		Metabolic function	
		**Mantel's r**	***p*_value**	**Mantel's r**	***p*_value**
Soil chemistry properties	pH	0.219	0.056	0.237	0.054
	OM	0.293	0.038*	0.431	0.005**
	NO_3^-–N	0.405	0.011*	0.336	0.015*
	NH_4^+–N	0.429	0.008**	0.352	0.013*
	AK	0.406	0.012*	0.338	0.016*
	AP	0.420	0.009**	0.344	0.014*
Soil enzymes	SUE	0.375	0.012*	0.274	0.037*
	SNR	0.414	0.011*	0.340	0.018*
Leaf physiological index	LS	0.220	0.057	0.057	0.306
	LMA	0.420	0.009**	0.158	0.115
	Pn	0.387	0.009**	0.171	0.109
	SPAD value	0.239	0.052	0.321	0.021*
Yield		0.110	0.217	0.423	0.003**

Note: LS, leaf size; LMA, leaf mass per area; Pn, net photosynthetic rate.

Table S3. Experimental design and scheme of the fertilization treatments.

	Treatment	Nutrient dosage (kg ha^{-1})			Fertilizer dosage (kg ha^{-1})			
		N	K_2O	P_2O_5	Urea (46% N)	SCNF (37% N)	KCL (60% K_2O)	$Ca(H_2PO_4)_2$ (12% P_2O_5)
Normal atmospheric environment (A)	A_CK	225	120	100	489	/	200	833
	A_S	225	120	100	/	608	200	833
elevated ambient O_3 concentration (E)	E_CK	225	120	100	489	/	200	833
	E_S	225	120	100	/	608	200	833

Note: SCNF, sulfur-coated controlled-release nitrogen fertilizer.
